# Comprehensive evaluation and clinical implementation of commercially available Monte Carlo dose calculation algorithm

**DOI:** 10.1120/jacmp.v14i2.4062

**Published:** 2013-03-04

**Authors:** Aizhen Zhang, Ning Wen, Teamour Nurushev, Jay Burmeister, Indrin J. Chetty

**Affiliations:** ^1^ Department of Radiation Oncology Gershenson Radiation Oncology Center, Wayne State University School of Medicine Detroit MI; ^2^ Department of Radiation Oncology Henry Ford Health System Detroit MI USA

**Keywords:** Monte Carlo, electron beam, commissioning, MU calculation

## Abstract

A commercial electron Monte Carlo (eMC) dose calculation algorithm has become available in Eclipse treatment planning system. The purpose of this work was to evaluate the eMC algorithm and investigate the clinical implementation of this system. The beam modeling of the eMC algorithm was performed for beam energies of 6, 9, 12, 16, and 20 MeV for a Varian Trilogy and all available applicator sizes in the Eclipse treatment planning system. The accuracy of the eMC algorithm was evaluated in a homogeneous water phantom, solid water phantoms containing lung and bone materials, and an anthropomorphic phantom. In addition, dose calculation accuracy was compared between pencil beam (PB) and eMC algorithms in the same treatment planning system for heterogeneous phantoms. The overall agreement between eMC calculations and measurements was within 3%/2 mm, while the PB algorithm had large errors (up to 25%) in predicting dose distributions in the presence of inhomogeneities such as bone and lung. The clinical implementation of the eMC algorithm was investigated by performing treatment planning for 15 patients with lesions in the head and neck, breast, chest wall, and sternum. The dose distributions were calculated using PB and eMC algorithms with no smoothing and all three levels of 3D Gaussian smoothing for comparison. Based on a routine electron beam therapy prescription method, the number of eMC calculated monitor units (MUs) was found to increase with increased 3D Gaussian smoothing levels. 3D Gaussian smoothing greatly improved the visual usability of dose distributions and produced better target coverage. Differences of calculated MUs and dose distributions between eMC and PB algorithms could be significant when oblique beam incidence, surface irregularities, and heterogeneous tissues were present in the treatment plans. In our patient cases, monitor unit differences of up to 7% were observed between PB and eMC algorithms. Monitor unit calculations were also preformed based on point‐dose prescription. The eMC algorithm calculation was characterized by deeper penetration in the low‐density regions, such as lung and air cavities. As a result, the mean dose in the low‐density regions was underestimated using PB algorithm. The eMC computation time ranged from 5 min to 66 min on a single 2.66 GHz desktop, which is comparable with PB algorithm calculation time for the same resolution level.

PACS number: 87.55.K‐

## I. INTRODUCTION

Electron beams are advantageous in the treatment of superficial tumors and frequently find applications in the treatment of head and neck cancers, chest wall irradiation for breast cancer, skin and lip cancers, and nodes boost. Accurate dose computation is essential for radiotherapy treatment to be successful. In current routine practice, electron beam monitor units (MU) are normally calculated manually or using commercial software with entered and derived data. The pencil beam (PB) algorithm is often used to calculate and visualize isodose distributions. One major limitation of the PB algorithm is its inaccuracy in predicting the dose in a variety of clinical situations such as perturbations by air cavities or other heterogeneities, as well as the backscatter from high‐density materials such as bone.^(^ ^1^ ^–^ ^5^ ^)^ Due to the limitations of the PB algorithm, electron beam isodose calculations are rarely performed in some institutions. Therefore, the adequate coverage of the target is not guaranteed and the actual dose delivered to the normal tissue is unknown. It is widely accepted that Monte Carlo simulations are an accurate method for calculating dose distributions in radiation therapy, provided the radiation source and phantom are accurately modeled and a sufficient number of particle histories are simulated.^(^ ^6^ ^–^ ^15^ ^)^ The superior accuracy of several Monte Carlo codes such as EGSnrc, PENELOPE, and DPM has been demonstrated for a wide range of materials and energies.^(^ ^16^ ^–^ ^21^ ^)^ However, the routine use of Monte Carlo simulations for external beam radiotherapy has been impeded by the long calculation time. This limitation has prompted researchers to improve the efficiency of their Monte Carlo dose engines. Parallel programming of PENELOPE and DPM codes have been implemented to reach high computation performance.^(^ ^22^ ^–^ ^24^ ^)^ Several Monte Carlo variance reduction techniques,^(^ ^25^ ^–^ ^27^ ^)^ such as voxel‐based Monte Carlo algorithm (VMC)^(^ ^25^ ^)^ and macro‐Monte Carlo (MMC) method,^(^ ^27^ ^)^ have also been introduced to increase calculation speed while preserving accuracy. The utilization of computer resources and variance reduction techniques makes Monte Carlo algorithm more practical for clinical situations.

A commercial Monte Carlo‐based dose calculation algorithm has become available for electron beam treatment planning in the Varian Eclipse treatment planning system (Varian Medical Systems, Palo Alto, CA).^(^ ^28^ ^)^ The electron Monte Carlo (eMC) algorithm employed by Eclipse is a fast implementation of the MMC method for calculation of dose from high‐energy electron beams. The MMC uses the MC technique, but is very different from the standard simulation of radiation transport. The MMC method has a database of probability distribution functions (PDFs) which are precalculated employing EGSnrc code system. The precalculations simulated the transport of incident electrons of various energies through small spheres of varying sizes and materials likely to be needed for actual MMC calculation. The CT absorber volume is preprocessed into many appropriate spheres with certain densities. Thus, the MMC step for primary particles is reduced essentially to a look‐up table process. Other approximations and techniques for the secondary particles are also made to improve the calculation speed.

There have been studies to evaluate the eMC algorithm in a homogeneous water phantom and heterogeneous phantoms. Popple et al.^(^ ^29^ ^)^ investigated the effect of grid size, accuracy, and smoothing level on the performance of the eMC algorithm for several phantom geometries. The accuracy of the eMC algorithm in a water phantom and heterogeneous phantoms was evaluated by Ding et al.^(^ ^30^ ^)^ who also addressed dose reporting issue. Hu et al.^(^ ^31^ ^)^ presented their experience of commissioning the eMC algorithm for four linear accelerators. The accuracy of the eMC algorithm for small circular cutouts in a water phantom was reported by Xu et al.^(^ ^32^ ^)^ The original eMC implementation was modified by Fix et al.^(^ ^33^ ^)^ to improve the accuracy of the dose calculation for low‐energy electron beams (4 MeV and 6 MeV). Aubry et al.^(^ ^34^ ^)^ validated eMC algorithm using radiochromic films and EGSnrc as a reference Monte Carlo algorithm. Despite these studies, current practice has not yet adopted eMC calculations for electron beam planning, and there has been no work to investigate the implementation of this algorithm in clinical practice. In this paper, we present our experience of commissioning, verification and clinical implementation of this eMC algorithm in Eclipse. The accuracy of the eMC algorithm was verified in a water phantom, heterogeneous solid water phantoms, and an anthropomorphic phantom. Clinical implementation of eMC algorithm was investigated by performing treatment plan calculations for 15 patients who underwent electron radiation treatment at our institution. In addition, dose calculation accuracy was compared between eMC and PB algorithms in heterogeneous solid water phantoms and an anthropomorphic phantom. Dose distributions for the 15 patients with various energy beams and applicator sizes were also calculated using both PB and eMC algorithms, for comparison.

## II. MATERIALS AND METHODS

### A. Eclipse eMC beam modeling

The eMC algorithm employed by Eclipse consists of two models: 1) transport model, MMC method calculating the dose deposited at each point, and 2) initial phase space (IPS) model describing the electrons that emerge from the treatment head of the linear accelerator.

The transport model of the eMC algorithm is an implementation of the local‐to‐global Monte Carlo (LTG MC) method. First, the probability distribution functions (PDFs) were generated in extensive precalculations by employing the EGSnrc code system in well‐defined local geometries. Then in a global geometry, absorber‐specific MC calculations are performed based on the PDFs generated in the local calculations. Calculation time is therefore reduced by substituting microscopic steps through the patient with fewer precomputed steps through macroscopic spheres.

The IPS model is based on precalculated data released from Varian for a machine type and configured using measured beam data. The precalculated data includes: a set of 50 depth‐dose curves for monoenergetic electrons calculated with MMC (0.5–25 MeV in 0.5 MeV steps) for all possible applicators and for two focus positions (10 cm and 50 cm), depth dose curves for edge electrons produced by monoenergetic target electrons for all possible energies and applicators, depth‐dose curves for transmission photons for all possible energies and applicators, and depth‐dose curves for main photons calculated with MMC for all possible energy modes and all applicators. The machine type parameters, such as focus position of main diverging beam and cutout material, are used for creating precalculated data in the IPS model. The configuration of the eMC algorithm for each beam energy requires measurements for all open field and applicator combinations. The open‐field measurements (without the applicator, with collimator jaws wide open) consist of the following: i) depth‐dose curves in water at source‐to‐surface distance (SSD)=100 cm and absolute dose at the calibration point on the depth‐dose curve, and ii) dose profiles in air at source‐to‐detector distance equal to 95 cm. The in‐air profiles provide direct electron fluence information needed to configure the incident beam characteristics. The applicator measurements are for each energy/applicator combination: depth‐dose curves in water at SSD=100 cm and absolute dose at the calibration point on the depth‐dose curve.

Required beam data for the eMC algorithm configuration were collected for beam energies 6, 9, 12, 16, and 20 MeV and applicator sizes 6×6(A06),10×10(A10),15×15(A15),20×20(A20), and $25\times 25\;{\text{cm}}^{2}$ (A25) available in the Eclipse treatment planning system. A Wellhofer Blue Phantom scanning system and a Wellhofer CC13 cylindrical ionization chamber (active $\text{volume}=0.13\;{\text{cm}}^{3}$) (IBA Dosimetry America, Bartlett, TN) were used to acquire the incident beam data for a Varian Trilogy linear accelerator (Varian Medical Systems).

The eMC algorithm has six user‐selectable parameters for individual calculations: calculation grid size, accuracy, maximum number of particle histories, random generator seed number, smoothing method, and smoothing level. The calculation grid size defines the resolution of the dose calculation and possible values are 1 mm, 1.5 mm, 2 mm, 2.5 mm, and 5 mm. The term accuracy as used by the vendor is defined as the average statistical uncertainty in all voxels within the body contours with a dose greater than 50% of the maximum dose. The average statistical uncertainty $S_{50}$ is calculated using

(1) $$S_{50}=\frac{1}{N_{50}}\sum D_{ijk}>50\%D_{\max}\frac{\Delta D_{ijk}}{D_{\max}}$$

where $N_{50}$ is the number of voxels satisfying the condition $D_{ijk}>50\%D_{\max}$. The simulation is divided into $N_{batch}$ batches, each containing 10,000 particles. The minimum number of batches in simulation is 10. The statistical uncertainties $\Delta D_{ijk}$ were calculated using the following formula:

(2) $$\Delta D_{ijk}^{2}=\frac{〈D_{ijk}^{2}〉-{〈D_{ijk}〉}^{2}}{N_{batch}-1}$$

where $〈D_{ijk}〉$ is the average dose and $〈D_{ijk}^{2}〉$ is the average dose squared. The eMC has five levels of statistical uncertainty, 1%, 2%, 3%, 5%, and 8%. The calculation time is inversely proportional to the square of the statistical uncertainty.

The maximum number of particle history specifies the maximum number of particles to be transported in a calculation. Calculation stops once the set number of particles has been transported, even if the desired accuracy is not reached. If the value selected for the maximum number of particle histories is zero, the calculation does not terminate until specified accuracy is achieved. The number of particles used in the calculation is reported in the dose calculation log. The random generator seed number defines the random number sequence used in the particle generator. The most accurate results are obtained using the smallest grid size (1 mm) and the lowest statistical uncertainty (1%). However, for practical reasons, the choice will be limited by calculation time, which is proportional to the number of simulated particle histories. Throughout the study, the eMC calculation grid size used was 1.5 mm for 6 MeV and 9 MeV, 2 mm for 12 MeV and 16 MeV, and 2.5 mm for 20 MeV. The accuracy was set at 1%, and the number of particle histories was set to zero to ensure that the desired accuracy was achieved. The default random generator seed number 39916801 was used for all the calculations.

The eMC in Eclipse offers no smoothing and 2D Median and 3D Gaussian smoothing options. Both 2D Median and 3D Gaussian smoothing methods can be applied with three different smoothing levels (low, medium, and strong). The 2D Median dose smoothing method determines the value of a pixel by examining the pixel values in its neighborhood on a slice and taking the median of these values. The neighborhood is defined by the smoothing level. The 3D Gaussian smoothing is performed using standard 3D convolution methods. It convolves the dose distribution with a three‐dimensional Gaussian, the standard deviation of which is defined by the smoothing level. The standard deviation for medium level smoothing is equal to the grid size. The standard deviations for low and strong levels of smoothing are equal to half and 1.5 times of grid size, respectively. A study by Ding et al.^(^ ^30^ ^)^ showed that 2D Median smoothing can remove a real dose gradient in the calculated dose distributions in heterogeneous phantoms. Therefore, the 3D Gaussian smoothing option was chosen for this study. All phantom images used were obtained through CT scan and DICOM image transfer.

### B. Homogeneous water phantom

The configured eMC electron beam model was first used to calculate dose distributions for each energy/applicator combination without cutout in a homogeneous water phantom. To evaluate the eMC performance for small field sizes, calculations were also performed for electron beam energies 9 MeV and 16 MeV using standard $10\times 10\;{\text{cm}}^{2}$ applicator with various cutout sizes. These cutouts reduced the field size down from the open $10\times 10\;{\text{cm}}^{2}$ field size to sizes 2×2,3×3,4×4, and $5\times 10\;{\text{cm}}^{2}$. Initial eMC plans were created in Eclipse without normalization and 100 cGy were prescribed. The beam profiles and PDD curves were measured using Wellhofer Blue Phantom scanning system. For the open applicators, depth dose curves and dose profiles were measured with a CC13 chamber. For the small electron fields a CC01 (active $\text{volume}=0.012\;{\text{cm}}^{3}$) chamber was used to maintain adequate spatial resolution. Moreover, data were obtained in continuous scanning mode with the lowest scan speed to ensure the accuracy of the measurements. Measured data were exported from OmniPro (Iba Dosimetry America) in ASCII format. The measured and calculated depth dose curves and off‐axis dose profiles were normalized to 100% at the depth of the calculated maximum dose for comparison.

For each beam energy, calculations were performed over the range of possible values of grid size using 1% statistical uncertainty and maximum number of particle history 0. We found that further decrease of the chosen grid size specified in Material and Methods Section A lengthened calculation time and could not improve the results significantly. It is also found that medium and strong levels of smoothing yielded no significant difference (<0.5%) in the PDD and dose profile calculations, and the differences between low and medium levels of smoothing calculations reached 3%. Strong level 3D Gaussian smoothing method was used in the calculations.

### C. Heterogeneous phantoms

**Figure 1:**
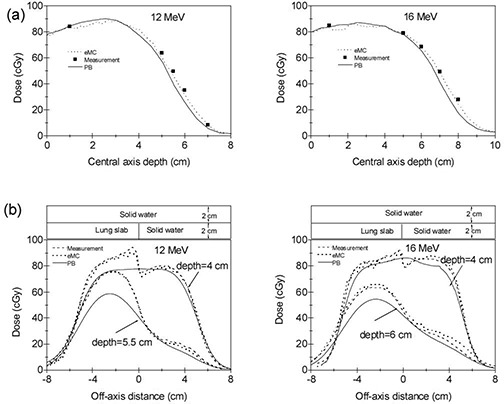
The PB and eMC calculations compared with measurements in the solid‐water phantom with lung slab at 100 cm SSD with $10\times 10\;{\text{cm}}^{2}$ applicator for energies 12 MeV and 16 MeV: (a) the CC01 ion chamber measured central axis depth dose curves compared with the calculations, and (b) the EDR2 film measured off‐axis dose profiles compared with the calculations.

**Figure 2:**
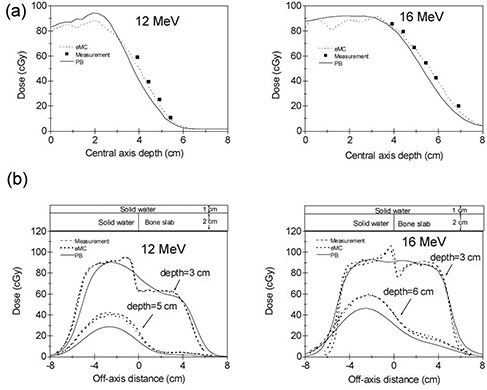
The PB and eMC calculations compared with measurements in the solid‐water phantom with bone slab at 100 cm SSD with $10\times 10\;{\text{cm}}^{2}$ applicator for energies 12 MeV and 16 MeV: (a) the CC01 ion chamber‐measured central axis depth dose curves compared with the calculations, and (b) the EDR2 film‐measured off‐axis dose profiles compared with the calculations.

Two solid water phantoms with lung and bone slabs (Gammex Inc., Middleton, WI) were used to verify the accuracy of the eMC algorithm in heterogeneous phantoms. The lung slab has a density of $0.3\;g\;{\text{cm}}^{-3}$ and the bone slab has a density of $1.8\;g\;{\text{cm}}^{-3}$. The dimension for both lung and bone slabs is 30×30×2 cm. Figure 1(b) shows the configuration of the solid water phantom with lung slab. Two cm solid water is followed by 2 cm lung slab and 2 cm solid water and then by 18 cm solid water. The vertical junction between the solid water and the lung slab is positioned on the central axis. The configuration of the solid water with bone slab only differs in that it contains 1 cm solid water above bone slab (as shown in Fig. 2(b). As in the case of homogeneous water phantom, eMC plans were created in Eclipse without normalization and 100 cGy were prescribed. Dose calculations using PB algorithm were also performed using eMC calculated monitor units (MUs). A CC01 ion chamber was used to measure point doses along the central axis for each irradiation condition. Dose profiles were measured with prepackaged EDR2 films placed at various depths. Film dosimetry was performed using a VIDAR scanner (VIDAR Systems/Contex Group, Stockholm, Sweden) and RIT software (Radiological Imaging Technology, Colorado Springs, CO). An optical density versus dose calibration curve was obtained for each film batch used in the study.

### D. Anthropomorphic phantom

**Figure 3:**
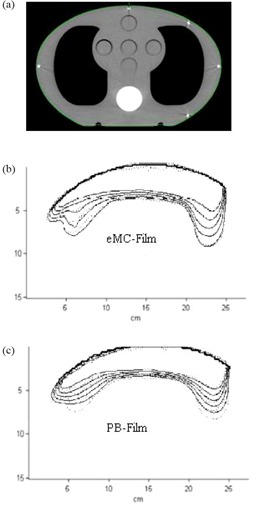
One CT transverse slice of the CIRS phantom (a); the eMC calculations compared with EDR2 film measurement (b); the PB calculations compared with EDR2 film measurement (c). Isodose lines of 80%, 70%, 60%, 50%, and 40% are depicted. The solid lines represent calculations and the dotted lines represent measurements.

To assess how accurately the eMC algorithm handle incident oblique beam in the presence of known inhomogeneity in a clinically relevant situation, an anthropomorphic CIRS phantom (model 002LFC, CIRS, Norfolk, VA) was used to evaluate the performance of the eMC algorithm. This phantom represents an average human thorax in density and structure, which consists of simulated lung, simulated bone, and water equivalent material. It has an elliptical shape and measures 30 cm long×30 cm wide×2 0 cm thick. Figure 3(a) shows one CT transverse slice of the CIRS phantom. The CIRS phantom was irradiated with a 9 MeV electron beam at a gantry angle of 15° and 100 cm SSD. The $20\times 20\;{\text{cm}}^{2}$ electron applicator was used and 100 MUs were delivered. Kodak EDR2 film (Eastman Kodak Company, Rochester, NY), cut to the same shape as the CIRS phantom, was placed in the central axis transverse plane. CT‐based treatment plans were calculated with 100 MUs using both eMC and PB algorithms to compare with measurement.

### E. Patient‐based anatomy

In order to evaluate the performance of the eMC algorithm in clinical situations, the commissioned eMC beam model was subsequently used to calculate dose distributions for 15 patients who underwent electron radiation treatment at Henry Ford Hospital. Among the 15 patients there is one chest wall case, two head and neck cancers, one metastatic melanoma of sternum case, one internal mammary nodes case, and ten breast boost cases. The treatment parameters employed for these patients cover all commissioned beam energies and applicator sizes. Details of the patient setup for treatment planning are listed in Table 1. For comparison purposes, the treatment plan for each patient was calculated using both eMC and PB algorithms.

**Table 1:** Details of the patient setup for treatment planning with SSD=100 cm. Also listed are the CPU times required for the plan calculation with the eMC algorithm.

*Patient*	*Energy (MeV)*	*Gantry Angle (°)*	*Applicator (cm* ^*2*^ *)*	*Bolus*	*Cutout*	*CPU Time (min)*
1 (neck)	9	16	15×15	No	Yes	30
2 (head)	12	0	15×15	Yes	Yes	14
3 (breast boost)	16	18	10×10	No	Yes	33
4 (breast boost)	12	310	10×10	Yes	Yes	64
5 (breast boost)	9	70	10×10	Yes	Yes	14
6 (breast boost)	12	0	10×10	No	Yes	23
7 (breast boost)	16	0	10×10	No	Yes	32
8 (breast boost)	12	314.5	15×15	No	Yes	32
9 (chest wall)	6	331.9	25×25	Yes	Yes	66
10 (melanoma in sternum)	9	0	20×20	No	Yes	30
11 (breast boost)	12	0	10×10	No	Yes	24
12 (internal mammary node)	20	345	6×6	No	Yes	5
13 (breast boost)	9	0	10×10	No	Yes	20
14 (breast boost)	16	26	15×15	No	Yes	60
15 (breast boost)	16	65	10×10	No	Yes	32

## III. RESULTS & DISCUSSION

### A. Homogeneous water phantom

**Figure 4:**
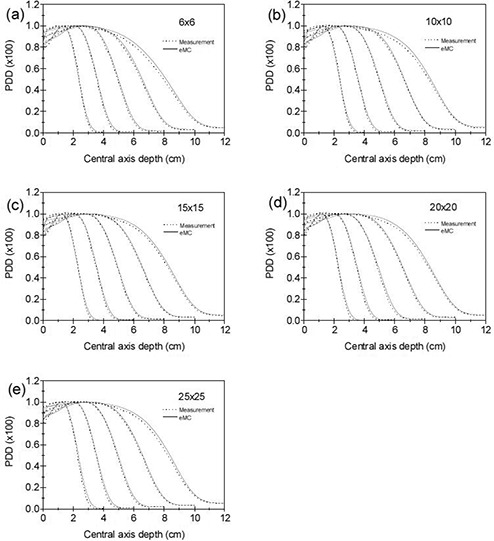
Central axis percent depth dose curve comparisons between eMC calculations and CC13 ion chamber measurements in water phantom for 6, 9, 12, 16, and 20 MeV electron beams (left to right) at 100 cm SSD: (a) 6×6 (b) 10×10 (c) 15×15 (d) 20×20 and (e) $25\times 25\;{\text{cm}}^{2}$ applicators. The measured and eMC calculated dose were normalized to 100% at the depth of the calculated maximum dose.

The comparisons between eMC calculated and CC13 ion chamber measured central axis percent depth dose (PDD) curves for applicator sizes of 6×6,10×10,15×15,20×20, and $25\times 25\;{\text{cm}}^{2}$ are shown in Figs. 4(a)–4(e) for beam energies 6, 9, 12, 16, and 20 MeV in a water phantom at 100 cm SSD. The PB algorithm calculation results are not presented here due to minor difference between PB and eMC calculations. The majority of the differences between measurements and eMC calculations are within 2%. For 20 MeV, larger differences of up to 3% are seen for all applicator sizes. Moreover, the measured PDD for 20 MeV follow the same trend (i.e., fall off more rapidly than eMC calculations).

**Figure 5:**
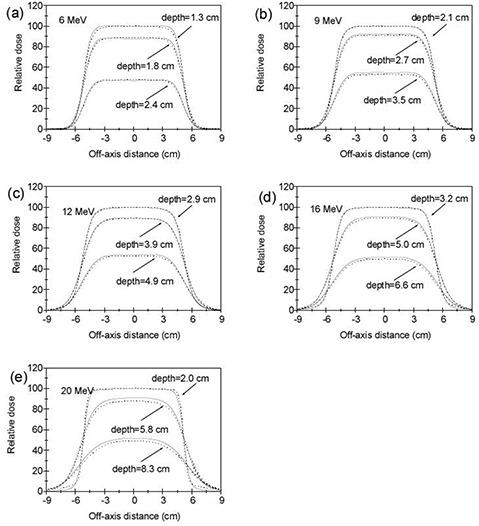
Cross‐plane dose profile comparisons at depths $d_{100}$, $d_{90}$ and $d_{50}$ between eMC calculations and CC13 ion chamber measurements in water phantom at 100 cm SSD with $10\times 10\;{\text{cm}}^{2}$ applicator: (a) 6 MeV, (b) 9 MeV, (c) 12 MeV, (d) 16 MeV, and (e) 20 MeV. The measured and calculated dose profiles were normalized to 100% at the depth of the calculated maximum dose on the central axis. The eMC calculations are represented by solid lines and the measurements are represented by dotted lines.

The dose profiles were measured and calculated at many depths for each energy/applicator combination. To save space, here we present only the results at depths $d_{100}$, $d_{90}$, and $d_{50}$ for each beam energy with the standard $10\times 10\;{\text{cm}}^{2}$ applicator. Figures 5(a)–5(e) show the comparisons between eMC calculated and CC13 ion chamber measured cross‐plane dose profiles at depths $d_{100}$, $d_{90}$, and $d_{50}$ for beam energies 6, 9, 12, 16, and 20 MeV at 100 cm SSD with $10\times 10\;{\text{cm}}^{2}$ applicator. Similar results were obtained between cross‐plane and in‐plane. In the high‐dose gradient region, the distance to agreement is within 2 mm for all beam energies. The agreement in the low‐dose low‐gradient region is within 2% for all beam energies. In the high‐dose low‐gradient region, differences between the measured and calculated dose profiles are within 2% except at depths $d_{90}$ and $d_{50}$ for 20 MeV, where the largest differences reach 3.5%.

**Figure 6:**
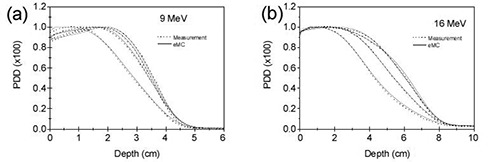
Percent depth dose comparisons between eMC calculations and CC01 ion chamber measurements in water phantom at 100 cm SSD using $10\times 10\;{\text{cm}}^{2}$ applicator with $4\times 4,3\times 3,2\times 2\;{\text{cm}}^{2}$ square cutouts (central axis) and half field block cutout (1 cm off‐central axis) for: (a) 9 MeV and (b) 16 MeV. The measured and eMC calculated depth dose were normalized to 100% at the depth of the calculated maximum dose.

**Figure 7:**
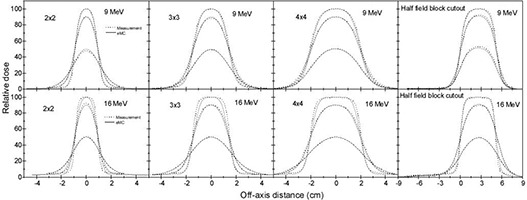
Cross‐plane dose profile comparisons at depths $d_{100}$, $d_{90}$ and $d_{50}$ between eMC calculations and CC01 ion chamber measurements in water phantom at 100 cm SSD using $10\times 10\;{\text{cm}}^{2}$ applicator with $4\times 4,3\times 3,2\times 2\;{\text{cm}}^{2}$ square cutouts and half field block cutout for beam energies 9 MeV and 16 MeV. The measured and calculated dose profiles were normalized to 100% at the depth of the calculated maximum dose on the depth doses.

Figures 6(a) and 6(b) show the percentage depth dose comparisons between eMC calculations and CC01 ion chamber measurements using $10\times 10\;{\text{cm}}^{2}$ applicator with $2\times 2,3\times 3,4\times 4\;{\text{cm}}^{2}$ square cutouts and half field block cutout ($5\times 10\;{\text{cm}}^{2}$) at 100 cm SSD for 9 MeV and 16 MeV electron beams, respectively. The differences between measurements and calculations are all within 3%. Cross‐plane dose profile comparisons at depths $d_{100}$, $d_{90}$, and $d_{50}$ with all cutout sizes are shown in Fig. 7. The agreement is within 3% or within 2 mm in regions with steep gradient for all cutouts and improves with increasing cutout size. In summary, between ion chamber measurements and eMC calculations PDD agree to 3% and dose profiles generally agree to 3%/2 mm. Larger discrepancies are observed in both PDD and dose profiles for high energy beam 20 MeV.

Xu et al.^(^ ^32^ ^)^ reported the PDD comparisons between eMC calculations and GAFCHROMIC EBT film measurements using $10\times 10\;{\text{cm}}^{2}$ applicator with 5, 4, 3, 2, and 1 cm diameter circular cutouts at 100 cm SSD for all beam energies. Their results showed that eMC calculations agreed well with EBT measurements for 5, 4, and 3 cm cutouts. For 2 cm cutout, large discrepancies were seen for higher energy beams (12–20 MeV), whereas our PDD and dose profile results show a 3%/2 mm agreement between eMC calculations and CC01 ion chamber measurements for both 9 MeV and 16 MeV beams. For 1 cm cutout, no discernible agreement between eMC calculations and EBT film measurements was found by Xu and colleagues. It should be noted that the small deviations in cutout size impact the dose distributions significantly for smaller cutouts, especially for 1 cm cutout. Considering the technical difficulties of making ideal 1 cm diameter cutout, direct comparison between eMC calculations and measurements is quite challenging. In addition, a field of this size is of little clinical significance.

### B. Heterogeneous phantoms

Experimental verification in heterogeneous slab phantoms was performed using an ion chamber and films placed at various depths. For each irradiation condition, the off‐axis dose profiles were measured at two different depths: right below the heterogeneity and deeper in the solid‐water phantom. Figure 1(a) shows the ion chamber measured and eMC and PB algorithms calculated central axis depth dose curves for two energies of 12 MeV and 16 MeV in solid water phantom with lung slab. The film measured and eMC and PB algorithms calculated off‐axis profiles are shown in Fig. 1(b). The results for the solid water phantom with bone slab are shown in Fig. 2. We note that the eMC calculation accurately predicts the impact of the adjacent inhomogeneity and the results agree well with ion chamber and film measurements at all depths. The differences between the measurements and eMC calculations can be attributed to uncertainty inherent in the film measurements and statistical uncertainty of the eMC algorithm. However, there are significant discrepancies at all depths between measurements and PB algorithm calculations. The PB algorithm is unable to predict the sharp dose increase and/or decrease close to the inhomogeneity. Thus, the PB algorithm may result in significant under‐ or overestimation of the dose in some cases, particularly those involving heterogeneities such as the lung–tissue and tissue–bone interfaces. Moreover, the PB algorithm significantly underestimates the dose at deeper depths beyond the inhomogeneities and produces errors up to 25%.

Ding et al.^(^ ^13^ ^)^ compared the PDD and dose profiles in heterogeneous phantoms between two commercial treatment planning systems. One uses a Monte Carlo dose calculation engine (Theraplan Plus v.3.8, MDS Nordion, Ottawa, Ontario, Canada) and the other uses a pencil beam algorithm (CADPLAN V6.27, Varian Medical Systems Inc., Palo Alto, CA). Our findings are consistent with the conclusions of Ding and colleagues that the PB algorithm results in large errors in phantoms containing three‐dimensional type inhomogeneities.

The observed small fluctuations visible in the eMC calculated curves in Fig. 1 and Fig. 2 are due to the fact that the calculations were performed without smoothing. We found that 3D Gaussian smoothing does not distort true dose profiles with the chosen grid sizes in this study. Thus, strong level 3D Gaussian smoothing method was used in the later eMC calculations for anthropomorphic phantom.

### C. Anthropomorphic phantom

Figure 3(b) shows the comparison between measured and eMC calculated isodose lines in the central axis plane of the CIRS phantom. Isodose lines of 80%, 70%, 60%, 50%, and 40% are depicted. It can be seen that eMC calculations agree well with film measurements at all isodose levels, although slight discrepancies in the 50% and 40% percent isodose lines in the right lung region can be observed. These discrepancies could be caused by image registration and measurement uncertainties.

Figure 3(c) shows the comparison between measured and PB calculated isodose lines in the central axis plane of the CIRS phantom. Although 80% and 70% isodose lines agree well between measurements and PB calculations, large discrepancies exist for 60%, 50%, and 40% isodose lines in the lung region. The discrepancies become more significant with increased depth in the lung part of the phantom plane. For 40% isodose line, penetration depth differences up to 0.7 cm and 1.0 cm are respectively observed in the right and left lung regions. Clearly, the electron range is underestimated if a pencil beam algorithm is used for dose calculation. For the low isodose lines, the significant differences in the lung region and much better agreement in the homogenous region of the CRIS phantom are consistent with the results shown in Fig. 1 and Fig. 2, where the PB calculations show large errors up to 25% at depths beyond the lung and bone slabs.

### D. Patient‐based anatomy

Treatment plans for 15 patients receiving electron radiation therapy at Henry Ford Hospital were calculated using both eMC and PB algorithms. The eMC calculations were performed on a 2.66 GHz desktop with no smoothing and all three levels of 3D Gaussian smoothing. Smoothing does not affect calculation time. The CPU time required for the plan calculations are listed in Table 1, which depends on the beam energy, applicator size, and the number of CT slices used to define the patient anatomy. Here, the longest time used for computation is 66 minutes for patient 9, where the smallest grid size 1.5 mm, 1.0 cm bolus, and the largest applicator $25\times 25\;{\text{cm}}^{2}$ were employed. We also find that the calculation time required is comparable between eMC and PB algorithms for the same resolution level.

The eMC algorithm in Eclipse can also be performed on multiple processors simultaneously, which is achieved using the distributed calculation framework (DCF).^(^ ^35^ ^)^ The Monte Carlo field parallelization factor in DCF determines into how many pieces each field is divided. Each piece uses different random number sequence and is processed on a different processor. The calculation time and the number of particles used in the simulation for each processor are reported in the dose calculation log. The implementation of eMC on parallel hardware results in a substantial speedup. For example, the 66 minutes computation time required for patient 9 can be reduced to a few minutes using seven processors.

**Figure 8:**
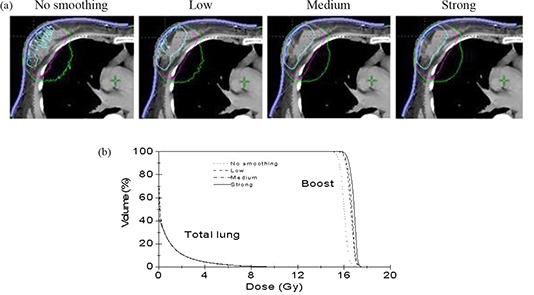
90%, 50%, and 30% isodose lines calculated by eMC (a) with no smoothing and low, medium, and strong levels of smoothing in central transverse plane for patient 4 (breast boost); DVHs comparison (b) for boost and total lung calculated by eMC.

In electron beam therapy, the dose is routinely prescribed to a certain isodose line, typically 90% isodose line. Table 2 shows the number of MUs per fraction calculated with PB algorithm and eMC algorithm with no smoothing and all three levels of 3D Gaussian smoothing based on this relative isodose line prescription. It is noted that the eMC calculated MUs consistently increases from without smoothing to strong level of smoothing. To see the effect of smoothing on dose calculations, we show in Fig. 8 a breast boost treatment plan (patient 4) calculated by eMC. Figure 8(a) shows 90%, 50%, and 30% isodose lines calculated with no smoothing and all three levels of smoothing (left to right). Compared with no smoothing the number of MUs increases by 3.6% with low level of smoothing applied, corresponding to a dramatic change in the appearance of 90% isodose line. Lots of discontinuities disappear and 50% and 30% isodose lines in the lung region also become smoother. No significant change is observed for all isodose lines from low to strong levels of smoothing, as there is no much change in the number of MUs. In the case of strong level smoothing, all isodose lines become very smooth. It can be seen that smoothing significantly affects isodose levels for high dose and has much less effect on isodose levels for low dose. Although isodose lines are greatly affected by the stochastic nature of eMC method, dose volume histograms (DVHs) were not sensitive to statistical uncertainty. That is, the fluctuation effect is not seen in DVHs. This is confirmed by the DVHs shown in Fig. 8(b). Compared with no smoothing, the mean dose to boost increases by 5% with strong level smoothing. As anticipated, DVHs for total lung are very similar in all cases. It can be concluded that 3D Gaussian smoothing can greatly improve the visual usability of isodose distributions and produces better target coverage.

**Table 2:** Number of monitor units per fraction for the patient treatment plans calculated by eMC and PB algorithms based on relative isodose line prescription method. The eMC calculations were performed with no smoothing and all three levels of 3D Gaussian smoothing. The lowest statistical uncertainty available in the treatment planning system (1%) was used.

*Patient*	*Smoothing*				*PB*
	*None*	*Low*	*Medium*	*Strong*	
	*eMC*				
1 (neck)	120	128	134	138	147
2 (head)	192	198	204	210	224
3 (breast boost)	204	208	215	221	224
4 (breast boost)	192	199	200	202	201
5 (breast boost)	189	192	195	198	199
6 (breast boost)	187	192	196	199	200
7 (breast boost)	176	179	183	188	201
8 (breast boost)	212	219	225	229	222
9 (chest wall)	195	203	204	213	220
10 (melanoma in sternum)	421	429	436	440	448
11 (breast boost)	193	199	205	208	212
12 (internal mammary node)	56	57	59	59	61
13 (breast boost)	186	189	192	194	199
14 (breast boost)	204	206	214	216	224
15 (breast boost)	192	200	207	214	222

In the cases of patients 3, 4, 5, 6, and 12, the eMC calculated MUs with strong level 3D Gaussian smoothing were close to the ones calculated by PB algorithm. However, other cases show different monitor units between PB and eMC algorithms. Significant monitor unit differences are observed for patients 1, 2, and 7 due to surface irregularities, obliquely incident beams, or the presence of heterogeneities in the close vicinity of target. In these cases, the PB calculated monitor units are approximately higher by 7% than that calculated by eMC algorithm with strong 3D Gaussian smoothing level.

**Figure 9:**
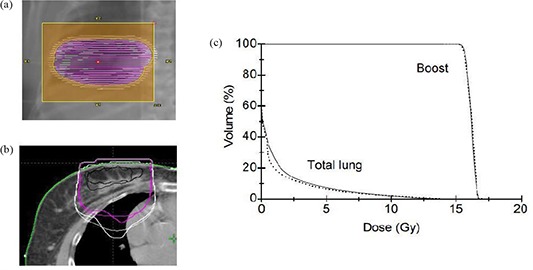
Treatment plan BEV display (a) for patient 7 (breast boost); comparison (b) of 90%, 50%, and 30% isodose lines in the central axis slice calculated by PB (thin line) and eMC (thick line) algorithms; DVHs (c) for boost and total lung calculated by PB (dotted line) and eMC (solid line) algorithms.

To compare the dose distributions calculated by eMC and PB algorithms, dose distributions were then calculated for each treatment plan using both eMC and PB algorithms with the same number of monitor units calculated by eMC strong level 3D Gaussian method. In the following we show calculation results for four representative patient cases: patient 7 (breast boost), patient 1 (head and neck cancer), patient 10 (metastatic melanoma in sternum), and patient 9 (chest wall boost). Figure 9(a) shows the treatment plan beam's eye view (BEV) for patient 7. The plan was generated using beam energy 16 MeV, gantry angle 0°, and applicator size $10\times 10\;{\text{cm}}^{2}$. The prescribed dose was 16.2 Gy. Figures 9(b) and 9(c) show the 90%, 50%, and 30% isodose lines in the central axis slice and DVHs calculated by PB and eMC algorithms, respectively. It can be seen (Fig. 9(c)) that the dose values in the target (boost) agree very well between PB and eMC algorithms, although 90% isodose lines show different shapes. Fifty percent and 30% isodose lines calculated by eMC algorithm penetrate deeper in right lung. Consequently, the mean dose to total lung is underestimated by 14% using PB algorithm.

**Figure 10:**
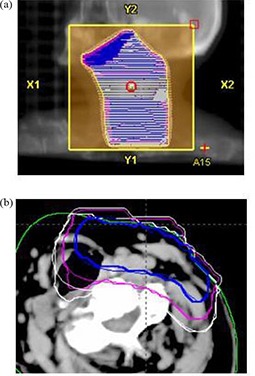
Treatment plan BEV display (a) for patient 1 (neck cancer); comparison (b) of 80%, 50%, and 30% isodose lines in the central axis slice calculated by pencil beam (thin line) and eMC (thick line) algorithms.

Figure 10(a) shows treatment plan BEV for patient 1. Beam energy 9 MeV, gantry angle 16°, and applicator size $15\times 15\;{\text{cm}}^{2}$ were used in the treatment plan; 33.25 Gy was prescribed to 90% isodose line in 25 fractions. Figure 10(b) shows 80%, 50%, and 30% isodose lines in the central axis slice calculated by PB and eMC algorithms. Penetration depth differences in the 50% and 30% isodose lines in the trachea reach 0.9 cm between eMC and PB algorithms, due to the limitations of the PB algorithm in accurately modeling electron scattering in the low density regions. The DVHs is not shown because target and critical structures were not contoured in this case.

**Figure 11:**
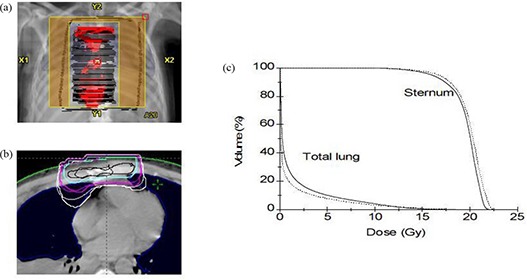
Treatment plan BEV display (a) for patient 10 (metastatic melanoma in sternum); comparison (b) of 90%, 80%, 50%, and 30% isodose lines in the central axis slice calculated by PB (thin line) and eMC (thick line) algorithms; DVHs (c) for sternum and total lung calculated by PB (dotted line) and eMC (solid line) algorithms.

Figure 11(a) shows treatment plan BEV for patient 10. The plan was generated using beam energy 9 MeV, gantry angle 0°, and applicator size $20\times 20\;{\text{cm}}^{2}$. A dose of 20 Gy was prescribed to 90% isodose line in 5 fractions. The comparison of 90%, 80%, 50%, and 30% isodose lines calculated with PB and eMC algorithms for the central axial slice is shown in Fig. 11(b). Figure 11(c) shows the DVHs comparison for the target (sternum) and critical structures (total lung). Notable differences are seen for 90%, 50%, and 30% isodose lines between PB and eMC calculations. Significant differences exist for 90% and 30% isodose lines. PB calculated 90% isodose line covers larger target (sternum) volume and eMC calculated 30% isodose line penetrates deeper in both lungs. As a consequence, PB algorithm shows 1.8% mean dose overestimation to the sternum and 35% mean dose underestimation to the lung relative to eMC algorithm. This demonstrates that the PB algorithm could not accurately predict the dose in regions with lung and bone inhomogeneous tissues.

**Figure 12:**
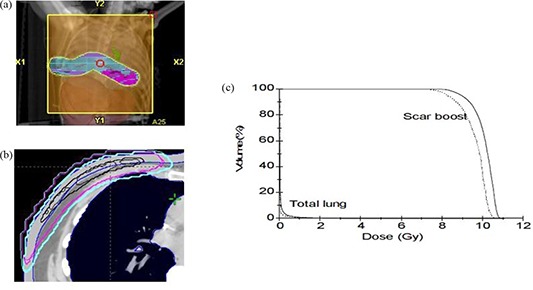
Treatment plan BEV display (a) for patient 9 receiving chest wall irradiation; comparison (b) of 90%, 50%, and 30% isodose lines in the central axis slice calculated by PB (thin line) and eMC (thick line) algorithms; DVHs (c) for the scar boost and total lung calculated by PB (dotted line) and eMC (solid line) algorithms.

Figure 12(a) shows the treatment plan BEV for patient 9, where beam energy 9 MeV, gantry angle 310°, 1 cm bolus, and applicator size $25\times 25\;{\text{cm}}^{2}$ were used. Ten Gy in 5 fractions was prescribed to 90% isodose line. Figure 12(b) shows the 90%, 50%, and 30% isodose lines in the central axis slice calculated by the PB and eMC algorithms. Although 50% and 30% isodose lines show good agreement between PB and eMC algorithms, 90% isodose line shows large discrepancy. Larger area is enclosed by eMC‐calculated 90% isodose line. The DVHs comparison for scar boost and total lung between eMC and PB algorithms is shown in Fig. 12(c). It is clear that PB DVH underestimates the dose in scar boost. Dose to total lung calculated by eMC is slightly higher than that calculated by PB algorithm. However, the dose values calculated by both algorithms are negligible because 1 cm bolus was used to shift the isodose lines towards the surface and reduce lung dose.

In the previous paragraphs, the monitor units were compared between different smoothing levels based on routine relative isodose line prescription. We found that the monitor units may change significantly at different smoothing levels due to dramatic change in isodose distributions. In order to avoid this relative effect, monitor units calculations were performed for each treatment plan when the dose was prescribed to the distal point of the region to be treated. Table 3 shows the monitor units calculated with PB algorithm and eMC algorithm with no smoothing and all three levels of 3D Gaussian smoothing based on point‐dose prescription. In contrast to previous findings, we noted that the monitor units do not change significantly at different smoothing levels. In addition, eMC and PB calculated monitor units are comparable in most cases. Large differences of 6.3%, 4.3%, and 4.2% were observed, respectively, for patients 9 (chest wall), 2 (head cancer), and 8 (breast boost) due to surface irregularities.

**Table 3:** Number of monitor units per fraction for the patient treatment plans calculated by eMC and PB algorithms based on point‐dose prescription method. The eMC calculations were performed with no smoothing and all three levels of 3D Gaussian smoothing. The lowest statistical uncertainty available in the treatment planning system (1%) was used.

*Patient*	*Smoothing*				*PB*
	*None*	*Low*	*Medium*	*Strong*	
	*eMC*				
1 (neck)	147	143	145	147	144
2 (head)	212	208	209	211	220
3 (breast boost)	243	241	240	241	240
4 (breast boost)	204	200	204	204	206
5 (breast boost)	273	275	274	275	273
6 (breast boost)	186	185	186	187	191
7 (breast boost)	190	191	193	194	194
8 (breast boost)	212	211	213	215	224
9 (chest wall)	204	204	206	208	221
10 (melanoma in sternum)	576	571	572	573	576
11 (breast boost)	203	205	204	205	206
12 (internal mammary node)	61	61	62	65	65
13 (breast boost)	184	184	184	184	188
14 (breast boost)	231	229	229	228	229
15 (breast boost)	241	242	242	243	247

## IV. CONCLUSIONS

This study presents a detailed and comprehensive evaluation of the eMC dose calculation algorithm in Varian Eclipse treatment planning system. The accuracy of the eMC algorithm was verified in a homogeneous water phantom (open fields and small fields), solid water phantoms containing both low‐ (lung) and high‐density (bone) materials, and an anthropomorphic phantom. Dose distributions were compared between measurements and calculations using percent depth dose, dose profiles at various depths, and isodose plots. We also compared the dose calculation accuracy between PB and eMC algorithms in the same treatment planning system. The eMC calculations generally agreed with the measurements to within 3% or 2 mm in both homogeneous and heterogeneous phantoms and large difference was observed for 20 MeV. However, the PB algorithm cannot predict the sharp dose gradients adjacent to the inhomogeneity and results in large errors (up to 25%) beneath the inhomogeneities such as bone and lung.

The investigation of clinical implementation of eMC algorithm was performed for 15 patients. Treatment plans for these patients were calculated using both PB and eMC algorithms with no smoothing and three levels of 3D Gaussian smoothing. Based on conventional electron beam therapy prescription method we found that the eMC calculated MUs consistently increases with increased level of smoothing. Three‐dimensional Gaussian smoothing can substantially improve isodose visualization and produces better target coverage. The calculated MUs and dose distributions between eMC and PB algorithms can be significantly different in the presence of oblique beam incidence, surface irregularities, and heterogeneous tissues. Monitor unit differences of up to 7% were observed between PB and eMC algorithms in our patient cases. For the purpose of comparison, monitor units calculations were also performed based on point‐dose prescription. The eMC algorithm showed increased penetration of electron beam into the low‐density regions such as lung and air cavities. Consequently, the mean dose in the low‐density region was underestimated using PB algorithm.

Based on our study, the eMC in Eclipse demonstrate clinically acceptable accuracy. With parallel computation, the dose distribution calculations can be completed in a few minutes to achieve 1% statistical uncertainty. The beam modeling requires minimum measured data. Thus, eMC algorithm provides an option for electron beam planning to ensure optimal target coverage and safe dose to critical organs. We hope this study can provide valuable guidance for the adoption of this commercial product in clinical use.
